# Catch-AF—Early Diagnosis of Symptomatic Arrythmias in the Waiting Period Prior to Seeing a Cardiologist in Victoria, British Columbia

**DOI:** 10.1016/j.cjco.2024.09.007

**Published:** 2024-09-25

**Authors:** Matthew W. Coxon, Kurt Hoskin, Martin van Zyl, Michael Thibert, Markus Sikkel

**Affiliations:** a Victoria Cardiac Arrhythmia Trials Inc., Victoria, British Columbia, Canada; b Division of Cardiology, University of British Columbia, Vancouver, British Columbia, Canada; c Division of Cardiology, Kelowna General Hospital, Kelowna, British Columbia, Canada; d Division of Cardiology, Royal Jubilee Hospital, Victoria, British Columbia, Canada; e Center for Cardiovascular Innovation, University of British Columbia, Vancouver, British Columbia, Canada; f Division of Medical Sciences, University of Victoria, Victoria, British Columbia, Canada

## Abstract

**Background:**

Atrial fibrillation (AF) is the most common cardiac arrhythmia. Given its often-paroxysmal nature, screening at a single time point, using a 12-lead electrocardiogram (ECG) or a Holter monitor, has limited benefit. The AliveCor KardiaMobile device is a validated ECG recorder that can be used for patient-directed arrhythmia diagnosis and symptom–rhythm correlation. The aim of this study was to evaluate whether using the KardiaMobile device could reduce the time-to-diagnosis, for AF as well as other arrhythmias. We hypothesized that providing patients with a KardiaMobile device during their waiting period for specialist care could reduce the length of time that passes before ECG detection of arrhythmia.

**Methods:**

Patients were randomized 1:1 to receive either standard monitoring (ECG and a Holter monitor) or enhanced monitoring (ECG, a Holter monitor, and a KardiaMobile device). Patients were instructed to upload ECG recordings if they had cardiac symptoms, so that symptom–rhythm correlation could be achieved. The primary outcome was the time-to-diagnosis for AF. The secondary endpoint was the time-to-diagnosis for any arrhythmias.

**Results:**

From October 2018 to October 2022, a total of 69 patients were enrolled, and they were followed up to 12 months. Overall, 6 of the 7 patients diagnosed with AF were in the enhanced-monitoring group (*P* = 0.106). The time-to-diagnosis was not significantly different in the 2 groups (*P* = 0.053). Overall arrhythmias were diagnosed in 10 patients (29%) in the standard-monitoring arm, compared to 22 patients (63%) in the enhanced-monitoring arm (*P* = 0.008). The time-to-diagnosis was reduced in the enhanced-monitoring arm (*P* = 0.010).

**Conclusions:**

The time-to-diagnosis of any arrhythmia was reduced significantly in patients randomized to receive KardiaMobile device monitoring. Providing patients with a KardiaMobile device may expedite the diagnosis of arrhythmias during the waiting period for specialist care.

**Clinical Trial Registration:**

NCT04302311.

## RÉsumÉ

**Contexte:**

La fibrillation auriculaire (FA) est l'arythmie cardiaque la plus courante. Étant donné sa nature souvent paroxystique, le dépistage à un seul moment, à l'aide d'un électrocardiogramme (ECG) à 12 dérivations ou d'un moniteur Holter, présente un avantage limité. L'appareil KardiaMobile d'AliveCor est un enregistreur ECG validé qui peut être utilisé pour diagnostiquer les arythmies à la demande du patient et établir une corrélation symptôme-rythme. L'objectif de cette étude était d'évaluer si l'utilisation de l'appareil KardiaMobile pouvait réduire le délai de diagnostic de la FA et d'autres arythmies. Nous avons émis l'hypothèse que le fait de fournir aux patients un appareil KardiaMobile pendant leur période d'attente pour des soins spécialisés pourrait réduire la durée avant la détection d'une arythmie par l'ECG.

**Méthodes:**

Les patients ont été randomisés 1:1 pour recevoir soit une surveillance standard (ECG et moniteur Holter), soit une surveillance améliorée (ECG, moniteur Holter et appareil KardiaMobile). Les patients ont reçu l'instruction de télécharger les enregistrements ECG s'ils ressentaient des symptômes cardiaques, afin de pouvoir établir une corrélation entre les symptômes et le rythme. Le critère d'évaluation principal était le délai de diagnostic de la FA. Le critère d'évaluation secondaire était le délai de diagnostic de toute arythmie.

**Résultats:**

D'octobre 2018 à octobre 2022, un total de 69 patients ont été inscrits et ils ont été suivis jusqu'à 12 mois. Dans l'ensemble, 6 des 7 patients diagnostiqués avec une FA étaient dans le groupe de surveillance améliorée (*p* = 0,106). Le délai de diagnostic n'était pas significativement différent dans les deux groupes (*p* = 0,053). Dans l'ensemble, des arythmies ont été diagnostiquées chez 10 patients (29 %) dans le groupe de surveillance standard, contre 22 patients (63 %) dans le groupe de surveillance améliorée (*p* = 0,008). Le délai de diagnostic a été réduit dans le groupe de surveillance améliorée (*p* = 0,010).

**Conclusions:**

Le délai de diagnostic de toute arythmie a été réduit de manière significative chez les patients randomisés pour recevoir une surveillance de l'appareil KardiaMobile. Le fait d'équiper les patients d'un appareil KardiaMobile peut accélérer le diagnostic des arythmies pendant la période d'attente des soins spécialisés.

Atrial fibrillation (AF) is the most common cardiac arrhythmia, and the likelihood of developing it increases as one’s age increases.^1^ The length of the waiting period to see a cardiologist in British Columbia in 2022 was approximately 16.4 weeks, according to the Fraser Institute.^2^ In our office in Victoria, British Columbia, the waiting period to see a cardiologist is longer, averaging 22 weeks currently. For patients with undiagnosed AF, this length of time increases their risk for developing thromboembolic complications in the absence of anticoagulation treatment.^3^ Patients with other undiagnosed arrhythmias may later face a significant emotional burden, owing to their having not known the severity or nature of the arrhythmia for an extended period of time.^4^

The KardiaMobile device (AliveCor, Mountain View, CA) is a validated, single-channel electrocardiogram (ECG) recorder that can be paired with a smartphone that has a high level of sensitivity (0.82-0.91) and specificity for AF diagnosis (0.97-0.99).^5^, ^6^, ^7^ The KardiaMobile can be used to empower patients to assess for arrhythmias at the time of symptom onset and can allow for symptom–rhythm correlation, with a 30-second single-lead ECG strip that can be sent readily to a physician. Given that embracing the use of remote technologies can be preferable for both healthcare personnel and patients, finding alternative methodologies, such as the KardiaMobile, is imperative.^8^ Additionally, the waiting period for specialist consultation often is wasted diagnostic time.^9^ Technologies such as the KardiaMobile, however, have the potential to transform the waiting period into a useful time for gathering data that can lead to a definitive diagnosis.^10^^,^^11^ The current prospective randomized study was conducted to demonstrate and quantify the usefulness of the KardiaMobile in diagnosing AF and other arrhythmias during the waiting period, prior to when a patient is seen by a cardiologist.

## Methods

### Study design and patient population

**Figure 1:**
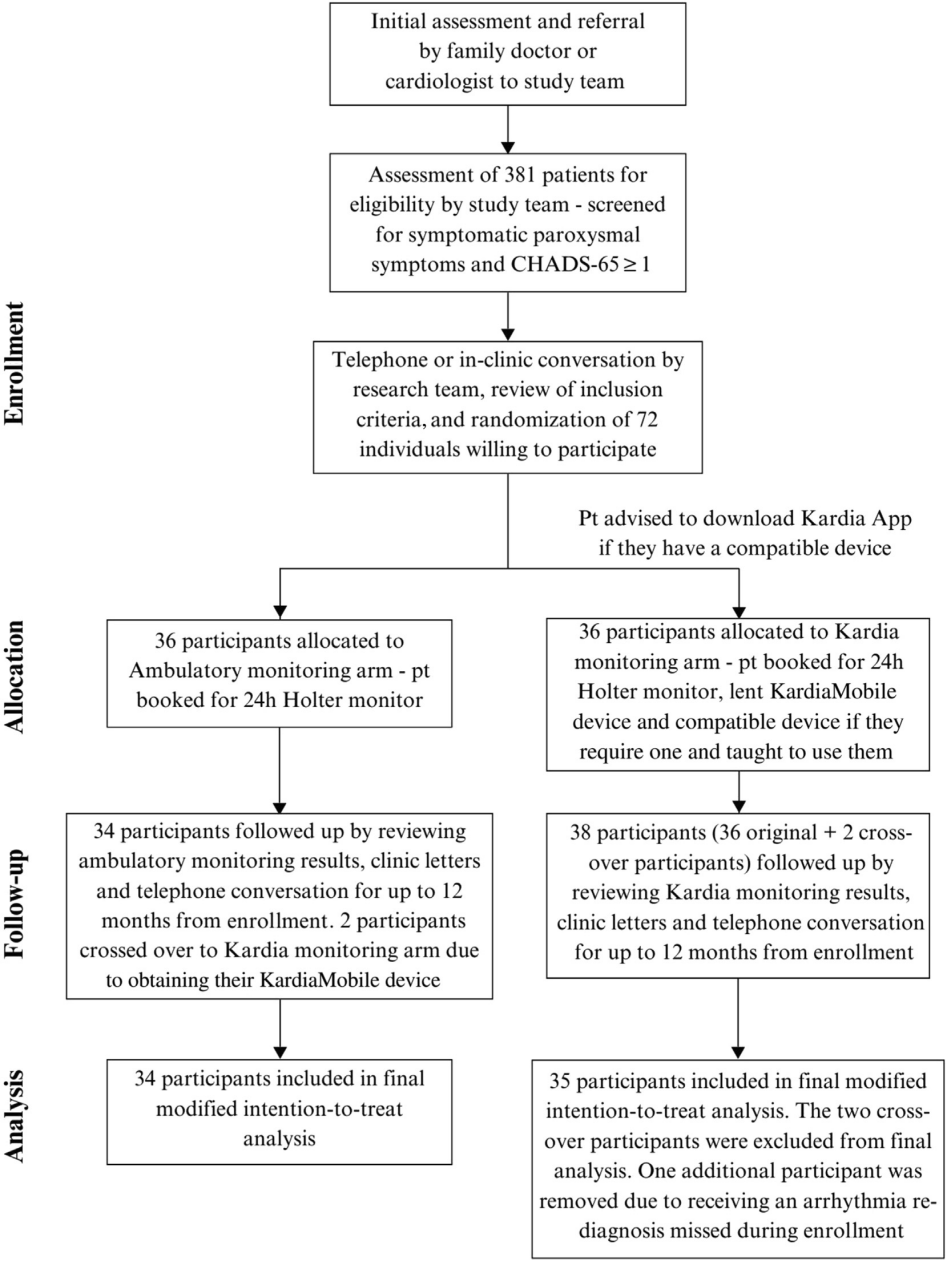
Patient flow diagram for the CATCH-AF study.

This randomized controlled study included patients referred to an outpatient cardiology office in Victoria, from October 2018 to October 2022. Patients were included if they has a history of symptomatic paroxysmal symptoms that could be attributable to a cardiac arrhythmia, including palpitations, dyspnea, or presyncope, and ≥ 1 risk factor per the Congestive Heart Failure, Hypertension, Age 65 years, Diabetes, Stroke/Transient Ischemic Attack (CHADS-65) algorithm (Fig. 1). This algorithm is a widely recognized method of assessing stroke risk; it assigns 1 point to congestive heart failure, hypertension, diabetes, and age ≥ 65 years, and 2 points for previous stroke and/or transient ischemic attack.^5^ Patients with known AF, cardiac symptoms typical of a nonarrhythmic cause, and/or those on anticoagulation therapy for an alternative reason, were excluded (Fig. 1). This study complied with the Declaration of Helsinki and was approved by the institutional ethics review board. Informed consent was obtained by the research team.

### Patient recruitment

At the beginning of the study, information regarding the study was distributed to various clinics in the community, which referred patients to the study site. Participants were then recruited, based on either the referral from a physician in the community or assessment by coordinators based at the clinic. In cases of physician referral, patients referred for palpitations or other symptoms of a potential arrhythmia also were asked about participating in the trial. If they were interested, their information was given to the coordinators, who then provided the potential participants with additional information. Coordinators also screened incoming patients who had not yet seen a cardiologist but who had received a referral to be provided with a Holter monitor, for symptoms corresponding to a potential arrhythmia. In some cases, cardiologists at the clinic referred patients to the study after completing a consultation that led to a diagnosis that could not have been obtained via other methods. From this process, a total of 381 patients were screened and assessed for eligibility. Participants who were interested and met the eligibility criteria then were sent requests to provide informed consent, and an appointment was arranged prior to their receipt of a Holter monitor. Although the study was powered for recruitment of 222 patients, achievement of this goal was hampered by a lack of continued funding for the study; as a result, only 72 patients were recruited.

### Intervention

Patients were randomized to receive either standard monitoring, which included use of a 12-lead ECG and a 24-hour Holter monitor, or enhanced monitoring, which included use of a 12-lead ECG, a 24-hour Holter monitor, and a KardiaMobile device. Randomization occurred in a 1:1 fashion, into either the standard-monitoring group or the enhanced-monitoring group, using a computer-generated program. Block randomization between the 2 arms was performed, using the online tool (“Create a blocked randomisation list” (Sealed Envelope) in blocks of 6. After a patient agreed to be randomized, their randomization was revealed via the software program, and the course of the next 6 months of their involvement in the trial was outlined for them. Patients who were randomized to the enhanced-monitoring group were provided with a KardiaMobile device, and an Android tablet (Samsung, San Jose, CA) if they did not have a compatible smartphone.

### KardiaMobile device and data collection

The KardiaMobile device used in the study is a small ECG device (dimensions: 8.2 x 3.2 x 0.35 cm; weight: 18 g) with which a user can collect diagnostic data by placing 2 fingers from each hand on the electrodes of the device. The KardiaMobile devices used in this study can connect with mobile devices, such as phones and tablets, through a wireless communication method, using ultrasonic audio; newer KardiaMobile devices utilize a Bluetooth connection. For data-collection purposes, devices were set to 30-second intervals allowing for a single-lead ECG to be transmitted from patient’s phones directly to the institutional database. Patients were educated on how to use the KardiaMobile app on their mobile device and upload ECG recordings to the institutional database. Patients were provided with a deidentified e-mail address and Kardia account, allowing them to upload recordings via their patient identification number, with no personal information being attached to their uploaded recordings. Patients were told to take ECG recordings if they had any cardiac symptoms, so that symptom–rhythm correlation could be achieved. The ECG recording is collected and analyzed on the smartphone, via the KardiaMobile algorithm, and it provides an initial classification of sinus rhythm, and an indication of possible AF, unclassified or unreadable. Upon receipt, the KardiaMobile ECG recordings were stored on a password-protected server, and printed for analysis. For all study patients, any recordings that were uploaded to the study team were further analyzed by a physician, regardless of the automated KardiaMobile device interpretation. Only physician-adjudicated KardiaMobile data were used in the data analysis. After analysis, KardiaMobile ECG recordings were scanned to the study team’s server and put into the patient’s chart, via the clinic’s electronic medical record system.

### Follow-up assessment

Patients in both study arms received follow-up assessment by the study team, either to 12 months or until an arrhythmia was detected. If an arrhythmia was detected, patients received an expedited cardiology consultation. At the end of the study period, patients were evaluated again, and their chart was reviewed to see if they had received a new diagnosis during their involvement in the trial. Patients in the enhanced-monitoring arm were instructed to return their KardiaMobile device if they had not received a diagnosis during their involvement in the study. Determination of the duration of the remainder of the follow-up period, and decisions to initiate use of any relevant medication, was left to the discretion of the consulting cardiologist. Although patients received follow-up assessment for up to 12 months, the original trial design stipulated that patients would receive follow-up assessment for up to 6 months. This provision was modified during the trial, as some patients mentioned, at their 6-month follow-up assessment, that they had an upcoming Holter monitor reading or had not been taking readings regularly (for those in the enhanced-monitoring arm). For this reason, the follow-up period was extended to include up to 12 months of assessment, and it included a telephone and chart review. With use of this method, patients were not lost to follow-up.

### Endpoints

The primary endpoint of this study was the detection of AF, on a 12-lead ECG, a Holter monitor, or a KardiaMobile device. The secondary outcome was the detection of any other arrhythmia, including supraventricular tachycardia (SVT) and ventricular ectopy.

### Statistical analysis

The participants’ information was uploaded to a secure and password-protected electronic spreadsheet on a password-protected server. Each participant’s baseline characteristics were recorded, including their date of birth, the number of **C**ongestive Heart Failure, **H**ypertension, **A**ge ≥ 75 years, **D**iabetes, **S**troke/Transient Ischemic Attack, **V**ascular Disease, **A**ge 65 to 74 years, **S**ex **C**ategory (CHA_2_DS_2_-VASc) risk factors they had, their medical history, any comorbid conditions, and their symptoms, episode assessments, and clinical assessments. Once each participant had completed the study, a summary of diagnosis (date, methodology, and type of, as well as time to) was analyzed, primarily in a descriptive manner. Continuous variables were defined using the median and interquartile range. Binary and categorical variables were summarized using percentages. Statistical analyses were conducted using Prism (GraphPad Software, Boson, MA), and a significance level of 0.050 (α) was used to compare group characteristics, via Fisher’s exact test, Kaplan–Meier analysis, and the log-rank test. Data analysis was completed using a modified intention-to-treat analysis, with participants being included in the final analysis only if they were followed per their originally assigned randomized arm.

## Results

During the study period, from October 2018 to October 2022, a total of 72 patients were enrolled, with 69 patients being included in the final, modified intention-to-treat analysis. Three participants were excluded from the final data analysis. For 2 of these patients, upon being randomized to receive the standard of care, they obtained a KardiaMobile device, outside the context of the study, and requested that they be allowed to send in transmissions of recordings. These patients eventually received a diagnosis through the study, but given that they were randomized to receive the standard of care but then bypassed study protocol and purchased a KardiaMobile device, they were excluded. The last patient was part of the enhanced-monitoring arm and was excluded, owing to previously having received a diagnosis of an arrhythmia, a point that was missed at the time of screening, owing to miscommunication with the patient. Given that this was a criterion for exclusion, and further arrhythmias were classified as a recurrence, data for this patient also were removed from the final analysis. Baseline characteristics for the final pool of patients and the 2 groups are outlined in Table 1. No significant difference occurred in the baseline characteristics between the 2 groups, with the exception of a higher frequency of obstructive sleep apnea occurring in the standard-monitoring group (n = 11 vs n = 3; *P* = 0.03).

**Table 1:** Summary of the baseline characteristics of the study participants

Baseline characteristics	Standard monitoring	Proportion of group, %	Enhanced monitoring	Proportion of group, %
Age, y, median (IQR)	69 (10.8)		68 (8.5)	
Average CHA_2_DS_2_VASc score	2.6		2.5	
CHF	2	5.90	0	0
HTN	15	44.10	21	60
Diabetes	6	17.60	3	8.60
Stroke and/or TIA	5	14.70	4	11.40
Vascular disease	3	8.80	4	11.40
CAD	3	8.80	5	14.30
OSA	11	32.40	3	8.60
Liver disease	0	0	2	5.70
Kidney dysfunction	1	2.90	3	8.60

Values are n, unless otherwise indicated. Apart from the incidence of obstructive sleep apnea (OSA) diagnosis, no significant differences occurred between the 2 arms.

CAD, coronary artery disease; CHA2DS2VASc, **C**ongestive Heart Failure, **H**ypertension, **A**ge ≥ 75 years, **D**iabetes, **S**troke/TIA, **V**ascular Disease, **A**ge 65 to 74 years, **S**ex **C**ategory; CHF, congestive heart failure; HTN, hypertension; IQR, interquartile range; TIA, transient ischemic attack.

**Figure 2:**
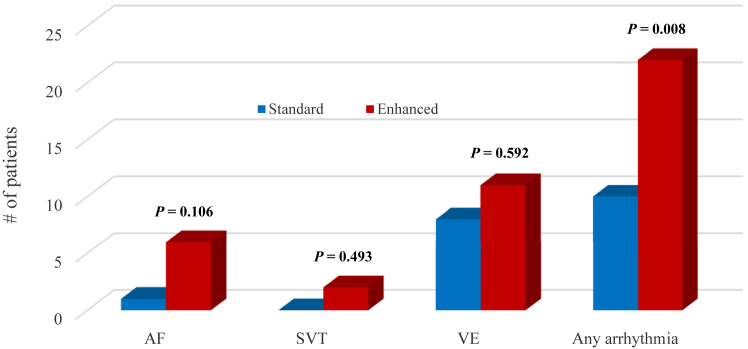
Number of arrhythmias detected using the Fisher’s exact test. A diagnosis was determined for 22 patients in the enhanced-monitoring arm (63%) vs 10 patients in the standard-monitoring arm (29%), demonstrating a significant difference between the 2 arms (*P* = 0.008). AF, atrial fibrillation; SVT, supraventricular tachycardia; VE, ventricular ectopy.

Overall, 7 of the 69 patients were diagnosed with AF, including 1 patient in the standard-monitoring group, and 6 patients in the enhanced-monitoring group. The primary outcome—time-to-diagnosis of AF—was not significantly different in the 2 arms (hazard ratio 6.19 [1.41-27.24], *P* = 0.053, log-rank test; 95% Confidence interval). A secondary analysis of the proportion of patients diagnosed in each arm also showed no significant difference (*P* = 0.106; Fisher’s exact test). In the enhanced-monitoring group, AF was detected with the KardiaMobile device for 5 of 6 patients (Fig. 2). For 5 of the 6 patients who received an AF diagnosis in the enhanced-monitoring arm, anticoagulation therapy was started within 2 weeks. Two patients in the enhanced-monitoring group were diagnosed with SVT on their KardiaMobile devices, and one underwent SVT ablation shortly thereafter. No patients were diagnosed with SVT in the standard-monitoring group (*P* = 0.493). Symptomatic premature ventricular contractions and premature atrial contractions were diagnosed in 8 patients in the standard-monitoring group, and 11 patients in the enhanced-monitoring group (*P* = 0.592). Overall arrhythmias, which included AF, SVT, and ventricular ectopy, as well as sinus tachycardia, were diagnosed in 10 patients (29%) in the standard-monitoring group, compared to 22 patients (63%) in the enhanced-monitoring group (*P* = 0.008).

**Figure 3:**
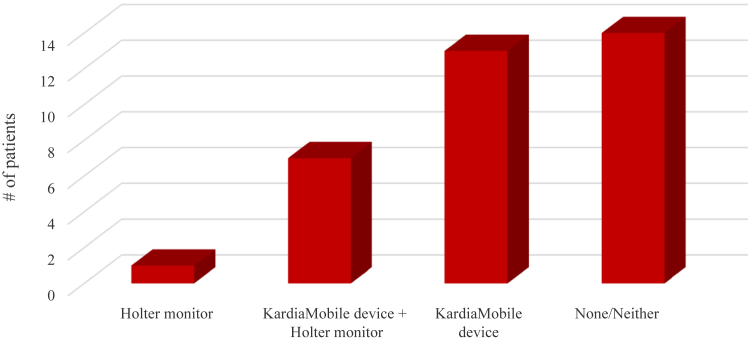
Comparison of the different methods for arrhythmia detection in the enhanced-monitoring arm. The most common method for arrhythmia detection was use of the KardiaMobile device alone, used by 13 of the total 35 patients in this arm.

Of the 35 patients within the enhanced-monitoring group, 13 (37%) received a diagnosis via the KardiaMobile device alone, whereas 7 patients (20%) received the same diagnosis from both their Holter monitor and their KardiaMobile device (Fig. 3). Of the other patients in the enhanced-monitoring group, 1 (3%) received a diagnosis solely from their Holter monitor, and the remaining 14 (40%) either received a diagnosis from a different source or never received a diagnosis. Within the enhanced-monitoring group, the mean number of transmissions submitted to receive a diagnosis was 11.97, with a median of 6. The number of transmissions submitted varied significantly, with a range of 0-93. Three participants did not submit any transmissions, with responses varying—from “no longer suffering from symptoms” to “symptoms being too infrequent to capture on the KardiaMobile device.”

**Figure 4:**
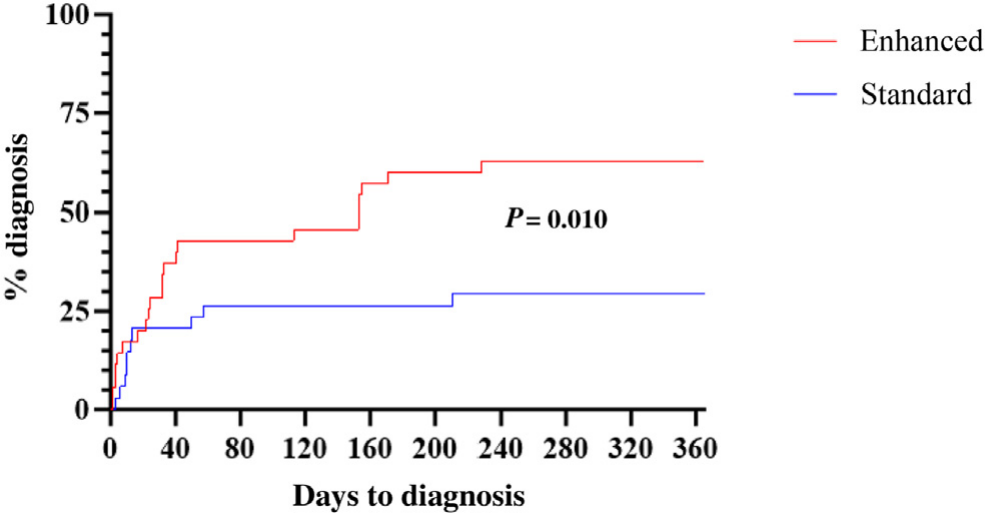
Comparison of the time-to-diagnosis for any arrhythmia. A significant difference was found between the two arms, with 22 patients in the enhanced-monitoring arm receiving a diagnosis within 12 months, vs 10 patients in the standard-monitoring arm. The time-to- diagnosis was found to be significantly different between the two arms (*P* = 0.010).

A comparison of the times-to-diagnosis in the 2 groups, for those with overall arrhythmias, showed that a diagnosis was received within 1 year for 29% of patients in the standard-monitoring group, vs 63% in the enhanced-monitoring group (Fig. 4). Analysis using the log-rank (Mantel–Cox) test demonstrates a statistically significant difference (hazard ratio 2.56 [1.28-5.12], *P* = 0.010) in the time-to-diagnosis between these 2 groups.

## Discussion

In this single-centre, prospective, randomized controlled trial, a trend occurred, toward a higher proportion of AF diagnoses being made among those using the KardiaMobile device, vs those using standard monitoring, although this difference was not at the level of statistical significance. We suspect this lack of significance is a result of the study being underpowered to detect such a difference—our original power calculations suggested the need to recruit 222 patients, but we were unable to do so because of a lack of funding.

The secondary outcome was time-to-diagnosis of any symptomatic arrhythmia. Here, we showed a significant improvement in time-to-diagnosis of any arrhythmia in patients who were randomized to the enhanced-monitoring arm, using Kaplan–Meier analysis. This improvement resulted in more patients being given a definitive diagnosis in the enhanced-monitoring arm of the study (22, vs 11 patients using standard monitoring).

In one instance, a patient was diagnosed with highly symptomatic SVT, and within 2 weeks underwent an SVT ablation after receiving expedited consultation. This example indicates that use of the KardiaMobile device during the waiting period to see a cardiologist may help in triaging patients, which could have a knock-on effect on healthcare utilization (eg, decreasing the number of emergency-room visits). In the enhanced-monitoring group, patients diagnosed with AF received rapid initation of anticoagulation therapy (on average, at 2 weeks after diagnosis), suggesting that use of the KardiaMobile device, during the waiting period for specialist care, could reduce the incidence of complications of AF, such as stroke.

We also provide some evidence that Holter monitoring itself may not have great diagnostic utility prior to consultation with a cardiologist. The fact that most patients given a diagnosis in this arm received it either solely from the KardiaMobile device or from both the KardiaMobile device and the Holter monitor^7^ provides support for utilizing the KardiaMobile device instead of a Holter monitor in certain circumstances. Rethinking regarding when a Holter monitor is utilized, and its replacement with a KardiaMobile device, may reduce not only the time-to-diagnosis but also the financial burden on the healthcare system, should such usage be financially efficient. Holter monitors often demonstrate a low diagnostic yield, and patients who are unable to receive a diagnosis are likely to seek out further healthcare services, either through emergency-room visits or future use of Holter monitors.^7^^,^^11^^,^^12^ Given that Holter monitoring has several known limitations, including the inaccessibility of the technology and the short duration of monitoring, which may be inadequate for patients with intermittent palpitations or symptoms, an alternative method for obtaining a diagnosis is desirable.

This study certainly suggests that healthcare spending could be better allocated, to provide patients with ambulatory ECG monitors, such as the KardiaMobile device, instead of paying clinics to provide Holter monitoring, in some circumstances. In our jurisdiction (British Columbia, Canada), the cost of Holter monitoring is reimbursed at approximately $140 CAD, including scanning and reading, whereas the costs of KardiaMobile devices and interpretations of their tracings are not reimbursed. In addition, although the KardiaMobile-device automated interpretation algorithm has a high level of specificity (97%) and a reasonable level of sensitivity (88%) for AF, it is not designed to give suggestions regarding diagnosis of other arrhythmias.^13^ All KardiaMobile tracings for this study were adjudicated individually by a physician. In relation to decision-making regarding use of anticoagulation therapy, automated algorithms are not yet considered reliable enough, per guidelines and consensus statements, to make a diagnosis of AF. In addition, the diagnoses for the 13 patients with non-AF arrhythmias would not have been classified correctly by the automated algorithm. This finding underscores the importance of having supporting physician interpretation of these tracings. The current billing model may require a reassessment, given the diagnostic utility shown by this study and others utilizing this type of technology.^6^^,^^10^^,^^14^^,^^15^

Another point worthy of note is that provision of the KardiaMobile device may be especially useful in healthcare systems such as ours that have prolonged waiting periods for specialist care. In fact, this type of provision of diagnostic equipment imbues the waiting period with diagnostic utility, as opposed to it being wasted time. Empowering patients to help collect data leading to their diagnosis, during the waiting period, also is likely to reduce patient frustration with the length of the waiting period. The average time-to-diagnosis in the enhanced-monitoring arm was 64 days (for the 22 patients who received a diagnosis), with patients often being seen shortly after a diagnosis was made. This trajectory helps in managing resources and triage appropriately in an under-resourced system, such as the Canadian healthcare system.

Even though we meant to utilize an intention-to-treat analysis, our final analysis excluded those patients who purchased a KardiaMobile device in response to being randomized to what they felt was the “wrong” arm (that is, the standard-monitoring arm). This process affected only 2 patients. Due to this exclusion, we utilized a modified intention-to-treat analysis. We acknowledge that this exclusion is an important limitation of our analysis, and alternative ways could have been used to deal with this situation.

Although the shortened time-to-diagnosis with the enhanced monitoring shows promise for this technology, we do not have evidence that this reduction is associated with improved patient outcomes. Within the scale of this study, however, we could not expect to show improved clinical outcomes, such as in stroke rate (which may decrease with more timely diagnosis of AF), as such studies usually require thousands of patients, and even then, often have negative outcomes.^16^ Instead, this study focuses more on how to use the inevitable waiting period to see a specialist most productively, particularly in the context of strained, publicly funded, healthcare systems.

Additionally, although the results of the enhanced-monitoring arm suggest that it is superior to Holter monitoring, this study was designed specifically for those with undiagnosed symptomatic palpitations; therefore, the shortened time-to-diagnosis applies to only those with symptomatic arrhythmias. Patients with asymptomatic arrhythmias, or prevalent arrhythmias (ie, persistent AF), may have other indications for a Holter monitor for which the KardiaMobile device is not useful. In addition, the KardiaMobile device proves most useful in the early detection of arrhythmias, prior to a formal diagnosis being made. Once a diagnosis is confirmed, Holter monitoring may provide comprehensive details of the arrhythmia, allowing for strategies to be developed for adequate rate or rhythm control; the KardiaMobile device has inferior performance in this regard.

One limitation of this technology lies in its accessibility, given that a smartphone or tablet, and savviness in using this technology, is required to utilize a KardiaMobile device. This lack of access is more applicable for the elderly population, which may have the most to gain from early AF detection and receipt of appropriate anticoagulation therapy. A limiting resource is physician interpretation of these data, and the potential to overwhelm healthcare workers with copious amounts of data. Some patients enrolled in this study could send up to 10 tracings a day, and although the time to analyze a KardiaMobile-device ECG usually is only 10-20 seconds, this volume puts a burden on the interpreter and may not involve the most efficient use of resources. From a practical standpoint, if the automated algorithm utilized to diagnose arrhythmias is improved in the future, the need for regular physician interpretation would be circumvented, at least part of the time. Additionally, medical staff can be trained to triage the KardiaMobile-device ECGs and recognize the most important ECGs for analysis, allowing physician interpretation to be utilized only when it is needed to make a final diagnosis. Lastly, recruitment for the study was hampered by a lack of continued funding, because of which the process was terminated early. Future work may yield more insightful findings, if the necessary number of participants can be recruited.

### Conclusion

This study shows that in patients with palpitations who are referred to cardiology services in jurisdictions in which a long waiting period is expected, the provision of a KardiaMobile device during the waiting period decreases the time-to-diagnosis for causes of palpitations. We also showed that conventional Holter monitoring has a very limited diagnostic yield in comparison. Although these findings are limited, due to early termination of the study, the use of the KardiaMobile device for monitoring during the waiting period certainly helped our clinic triage patients more appropriately.
