# Catch-AF—Early Diagnosis of Symptomatic Arrythmias in the Waiting Period Prior to Seeing a Cardiologist in Victoria, British Columbia

**DOI:** 10.1016/j.cjco.2025.03.005

**Published:** 2025-03-08

Author Corrections to “Catch-AF, Early Diagnosis of Symptomatic Arrythmias in the Waiting Period Prior to Seeing a Cardiologist in Victoria, British Columbia [CJC Open 6 (2024) 1476-1483]”

Matthew W. Coxon, MSc,^a^ Kurt Hoskin, MD,^b,c^ Martin van Zyl, MD,^b,d^ Michael Thibert, MD,^b,d^ and Markus Sikkel, MD, PhD ^b,d,e,f,^

^a^ Victoria Cardiac Arrhythmia Trials Inc., Victoria, British Columbia, Canada

^b^ Division of Cardiology, University of British Columbia, Vancouver, British Columbia, Canada

^c^ Division of Cardiology, Kelowna General Hospital, Kelowna, British Columbia, Canada

^d^ Division of Cardiology, Royal Jubilee Hospital, Victoria, British Columbia, Canada

^e^ Center for Cardiovascular Innovation, University of British Columbia, Vancouver, British Columbia, Canada

^f^ Division of Medical Sciences, University of Victoria, Victoria, British Columbia, Canada

The authors regret that the Funding Sources information was incomplete, and this should read:

The project was funded by Servier and the Victoria Hospitals Foundation via a Catalyst Grant awarded to M.S.

The authors apologise for any inconvenience caused.

