# Supplementary figures and images for: Catch-AF—Early Diagnosis of Symptomatic Arrythmias in the Waiting Period Prior to Seeing a Cardiologist in Victoria, British Columbia
Source: CJC Open. 2024 Sep 25;6(12):1476–83. doi: 10.1016/j.cjco.2024.09.007 (PMC11681358; doi:10.1016/j.cjco.2024.09.007)

Supplemental Appendix – Consort Diagram

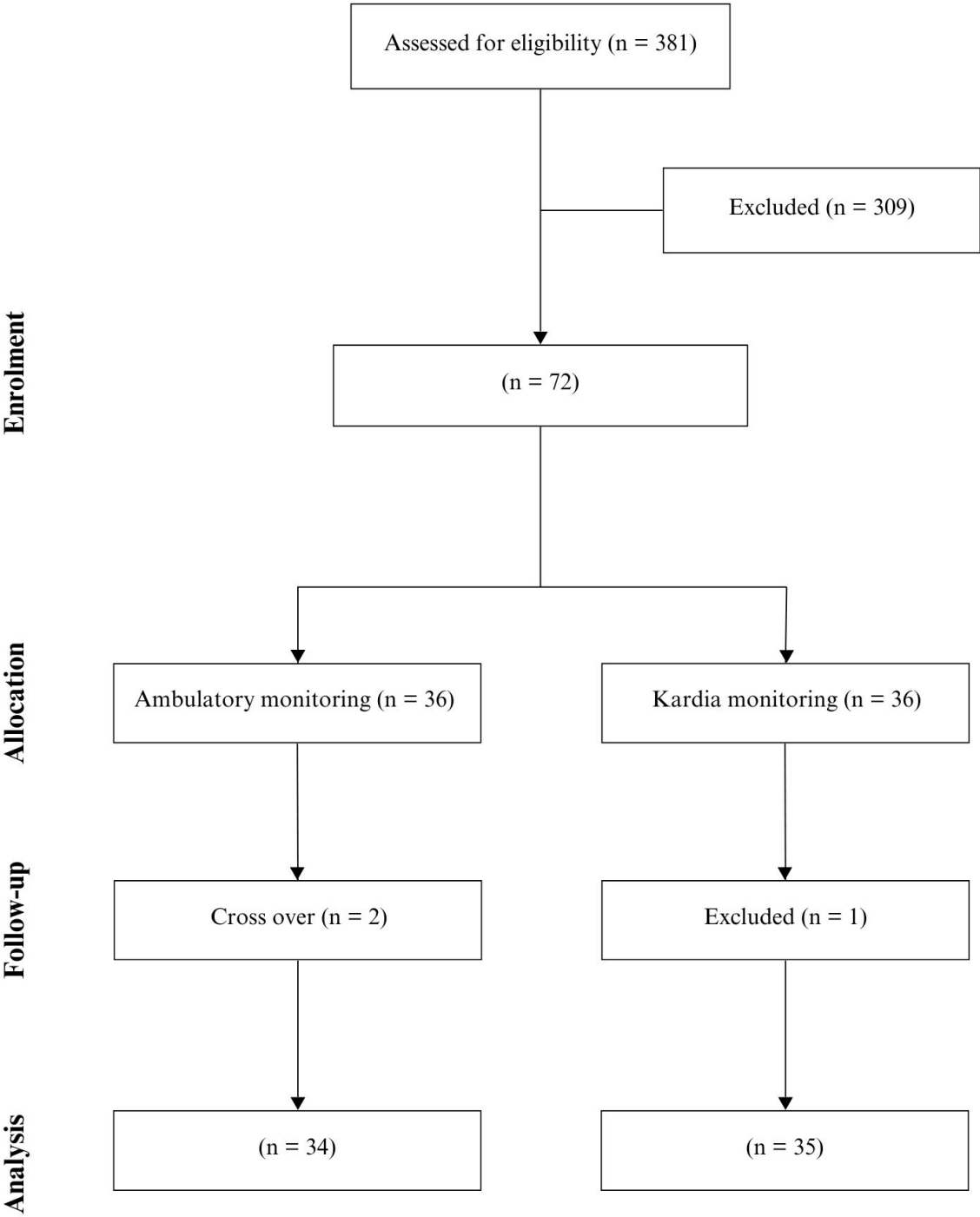

Supplement: Supplementary Appendix [file mmc1.pdf]
